# Supplementary material for: Statistics of antibody binding to the spike protein explain the dependence of COVID 19 infection risk on antibody concentration and affinity
Source: Sci Rep. 2022 Jun 7;12:9379. doi: 10.1038/s41598-022-13748-3 (PMC9172616; doi:10.1038/s41598-022-13748-3)
Supplement: Supplementary file 1 — Supplementary Information. [file 41598_2022_13748_MOESM1_ESM.docx]

**Supporting Information**

**Statistics of antibody binding to the spike protein explain the dependence of COVID 19 infection risk on antibody concentration and affinity**

David E Williams

School of Chemical Sciences, University of Auckland, Private Bag 92019, Auckland, 1142, New Zealand

**Calculation of the occupancy probability**

The occupancy probability, *p(s,t)* , is determined as follows ^1^:

[probability that occupancy of a particle is *s* at time *t* ] =

[probability that the occupancy was *(s-1)* at *(t-δt)* and that one further antibody was captured in the interval *(t-δt)* to *t* ]

+ [probability that the occupancy was *(s+1)* at *(t-δt)* and that one antibody was desorbed from the surface in the interval *(t-δt)* to *t* ]

— [probability that the occupancy was at *s* at *(t-δt)* and that antibody was captured or lost in the interval *(t-δt)* to *t* ]:

$p(s,t)=p(s-1,t-\delta t)\lambda_{1,s-1}\delta t+p(s+1,t-\delta t)\lambda_{2,s+1}\delta t+p(s,t-\delta t)(1-\lambda_{1,s}\delta t-\lambda_{2,s}\delta t)$

Hence, altering the notation : *p(s,t)* = *p_s_* , and substituting for *λ*

$$\frac{dp_{s}}{dt}=p_{s-1}\lambda_{1,s-1}+p_{s+1}\lambda_{2,s+1}-p_{s}\left( \lambda_{1,s}+\lambda_{2,s} \right)$$

$$=p_{s-1}k_{on}c\left( 1-\frac{s-1}{N} \right)+p_{s+1}k_{off}\left( s+1 \right)-p_{s}\left( k_{on}c\left[ 1-\frac{s-1}{N} \right]+k_{off}s \right)$$

 - (1)

For the state 0, from which there is no antibody desorption,

$$\frac{dp_{0}}{dt}=p_{1}k_{off}-p_{0}k_{on}c$$

 (2)

And for the state *N*, from which there is no further antibody adsorption,

$$\frac{{dp}_{N}}{dt}=p_{N-1}k_{on}c\left( 1-\frac{N-1}{N} \right)-p_{N}k_{off}$$

 (3)

The initial condition is: *p*(0) = 1 at *t* = 0 and furthermore $\sum_{s=0}^{N} p_{s}=1$ at any *t*.

The solution for the time-varying probabilities can be obtained numerically. The solution for the steady-state occupation probability is obtained by setting the derivatives to zero and solving recursively starting with the determination of *p*_1_ from eq(2), applying eq (1) to obtain successively the *p_s_*, and applying $\sum_{s=0}^{N} p_{s}=1$ to obtain *p*_0_.

Thus, defining $z=\frac{k_{on}c}{k_{off}}$ and $r_{s}=\left( \frac{1}{s!} \right)\left( \frac{N!}{N^{s}\left( N-s \right)!} \right)$ gives:

$p_{s}={r_{s}z^{s}}/{\sum_{s=0}^{N} r_{s}z^{s}}$ (3)

If *N* is very large and the total occupancy is sufficiently small, then *p_s_* follows a Poisson distribution: $p_{s}=\frac{z^{s}}{s!}\exp\left( -z \right)$.

1. Ishida, K., Stochastic Model for Langmuir Isotherm. *Bulletin of the Chemical Society of Japan* **1969,** *42* (2), 563-564.
